# Fanconi Anemia: Interplay Between DNA Repair Defects, Mitochondrial Dysfunction, and Oxidative Stress

**DOI:** 10.3390/cells15090753

**Published:** 2026-04-23

**Authors:** Giorgia Damonte, Matilde Balbi, Andrea Amaroli, Vanessa Cossu, Isabella Panfoli, Enrico Cappelli, Silvia Ravera

**Affiliations:** 1 Department of Experimental Medicine, University of Genoa, Via De Toni 14, 16132 Genova, Italy; giorgia.damonte@edu.unige.it (G.D.); matilde.balbi@unige.it (M.B.); silvia.ravera@unige.it (S.R.); 2 BIO-Photonics Overarching Research Laboratory (BIOPHOR), Department of Earth, Environmental and Life Sciences (DISTAV), University of Genoa, 16132 Genoa, Italy; andrea.amaroli@unige.it; 3 IRCCS Azienda Ospedaliera Metropolitana, Largo Rosanna Benzi 10, 16132 Genova, Italy; 4 Department of Pharmacy—(DIFAR), University of Genoa, Viale Benedetto XV 3, 16132 Genova, Italy; 5 Hematology Unit, IRCCS Istituto Giannina Gaslini, Via Gerolamo Gaslini 5, 16148 Genova, Italy; enricocappelli@gaslini.org

**Keywords:** Fanconi anemia, bone marrow failure, oxidative stress, mitochondrial dysfunction, antioxidant defenses, redox homeostasis, hematopoietic stem cells, antioxidant therapy

## Abstract

**Highlights:**

**What are the main findings?**

- In addition to its canonical DNA repair defect, Fanconi anemia is associated with mitochondrial dysfunction, oxidative stress, and impaired antioxidant responses across several experimental systems.
- Available preclinical studies and limited early clinical data suggest that modulation of redox balance and mitochondrial metabolism may improve cellular homeostasis in FA.

**What are the implications of the main findings?**

- Targeting oxidative stress and mitochondrial bioenergetics may represent a complementary therapeutic strategy alongside current genetic, hematologic, and supportive treatments for FA, although current evidence remains substantially stronger in primary and immortalized cellular models.
- Further studies and clinical trials are necessary to translate the effects of antioxidant molecules or oxidative metabolism modulators observed in vitro on the homeostasis of FA cells.

**Abstract:**

Fanconi anemia (FA) is a rare inherited disorder classically defined by defective DNA interstrand crosslink repair, leading to bone marrow failure and cancer predisposition. Increasing evidence indicates that FA pathophysiology extends beyond genomic instability to include mitochondrial dysfunction, oxidative stress, and impaired antioxidant responses. Across multiple cellular models and patient-derived samples, FA cells display altered mitochondrial bioenergetics, increased reactive oxygen species (ROS) production, and defective activation of redox-adaptive pathways, contributing to cumulative damage to DNA, lipids, and proteins. These alterations are particularly relevant in hematopoietic stem and progenitor cells, where metabolic stress and redox imbalance amplify stem cell exhaustion. Current data support a bidirectional interplay in which mitochondrial dysfunction and oxidative stress act mainly as secondary but amplifying factors of the primary DNA repair defect, establishing pathogenic feedback loops. Preclinical studies suggest that modulation of redox balance and mitochondrial function may improve cellular homeostasis, and early clinical investigations of antioxidant strategies indicate acceptable safety and measurable effects on oxidative biomarkers. However, clinical evidence remains limited and heterogeneous, with uncertain impact on long-term disease progression. Moreover, most mechanistic insights derive from in vitro or patient-derived models, while animal models and longitudinal clinical studies remain insufficient. Overall, a more integrated and translational framework is needed to clarify causality, validate biomarkers, and define the therapeutic potential of targeting metabolic and redox pathways in FA.

## 1. Fanconi Anemia: A Classic View

### 1.1. Overview of Fanconi Anemia

Fanconi anemia (FA) is a rare autosomal or X-linked recessive genetic disorder with an estimated incidence of 1–5 per million live births, representing one of the most common inherited bone marrow failure (BMF) syndromes [1]. FA is caused by germline mutations in several genes encoding proteins involved in a common DNA repair pathway specialized in resolving DNA interstrand cross-links (ICLs), which can hinder replication fork progression [1]. Since its first clinical description by Swiss pediatrician Guido Fanconi in 1927, our understanding of this complex disease has evolved substantially [2].

Although FA is fundamentally defined by defects in the DNA interstrand crosslink repair pathway, increasing evidence suggests that mitochondrial dysfunction, oxidative stress, and defective antioxidant responses may contribute to disease severity and progression [3,4,5,6,7].

Thus, the present review aims to clarify the contribution of mitochondrial dysfunction and redox imbalance to the etiopathogenesis of FA, describing the interplay between DNA damage, metabolic dysfunction, and redox imbalance, and to explore potential therapeutic implications.

### 1.2. Clinical Manifestations

The clinical presentation of FA is characterized by a distinctive triad of features: congenital abnormalities, progressive BMF, and markedly increased cancer susceptibility [1,8,9,10,11]. Typical phenotypic features include short stature, skeletal abnormalities such as radial ray defects and thumb anomalies, skin pigmentation changes including café-au-lait spots, microcephaly, and developmental anomalies affecting the kidneys, heart, and gastrointestinal tract. Many patients exhibit features consistent with VACTERL-H association (vertebral, anal, cardiac, tracheoesophageal fistula, esophageal/duodenal atresia, renal, limb anomalies, and hydrocephalus) and PHENOS features (pigmentation abnormalities, small head/eyes, neurologic and otologic anomalies, short stature). The severity and presence of these abnormalities depend on the specific gene mutations within the FA/BRCA DNA repair pathway and whether variants are null or hypomorphic. Patients with mutations in certain genes like *FANCB* or *FANCD2* tend to have more severe congenital malformations, including renal malformations and microcephaly. Overall, most patients with FA present with at least one physical abnormality involving multiple organ systems [12,13,14].

Progressive bone marrow failure represents the hallmark and primary cause of morbidity and mortality in FA patients, with approximately 80–90% developing aplastic anemia during their lifetime, typically manifesting during the first decade of life [15]. This hematologic deterioration results from the progressive depletion of hematopoietic stem and progenitor cells (HSCs/HPCs), a process that reflects the cumulative effects of chronic DNA damage, oxidative stress, and metabolic dysfunction on stem cell fitness and homeostasis [16].

FA-patients display a 500-fold increased risk of developing acute myeloid leukemia and myelodysplastic syndrome, with approximately 20–25% developing these myeloid malignancies [17].

Beyond hematologic manifestations, FA patients face a dramatically elevated cancer predisposition, with a cumulative incidence approaching 50% by age 50 [18,19]. This includes a 500–700-fold (some reports indicate over 1000-fold) increased risk of developing head and neck squamous cell carcinomas (HNSCC), which often arise at a younger age (median approximately 30 years) and demonstrate more aggressive behavior than in the general population [20]. Notably, HNSCC in FA patients can develop even in the absence of conventional risk factors such as tobacco or alcohol exposure, and frequently presents as multifocal, recurrent, and high-grade tumors with survival rates typically under two years [21,22]. Additionally, FA patients are predisposed to other solid tumors, including squamous cell carcinomas of the anogenital tract and skin [23,24].

### 1.3. Genetic Heterogeneity and Molecular Architecture

Despite a relatively homogeneous clinical and cellular phenotype across complementation groups, FA is genetically heterogeneous, with biallelic mutations in at least 23 genes (FANCA–FANCW), all of which encode proteins that participate in a common DNA damage response pathway essential for the repair of DNA interstrand crosslinks and the preservation of genomic stability [25,26]. The molecular architecture of the FA/BRCA pathway can be schematically organized into three functional modules: the upstream FA core complex, the FANCD2–FANCI (ID2) complex, and downstream repair effectors linked to homologous recombination [27,28,29,30,31,32]. The FA core complex includes eight canonical FA proteins—FANCA, FANCB, FANCC, FANCE, FANCF, FANCG, FANCL, and FANCM—together with several associated factors, and functions upstream to promote monoubiquitination of the FANCD2–FANCI complex, a key activation step in the pathway [33,34,35,36,37,38,39].

Activated ID2 then coordinates the recruitment of downstream repair proteins, including BRCA-related factors such as FANCD1/BRCA2, FANCN/PALB2, FANCJ/BRIP1, and FANCO/RAD51C, thereby enabling replication-associated repair of DNA crosslinks and maintenance of chromosomal stability [31,32,40,41]. Loss-of-function mutations in any component of this pathway result in failure of ID2 monoubiquitination and/or impaired homologous recombination repair, leading to the accumulation of DNA damage and genomic instability characteristic of FA [32,42,43].

### 1.4. The Cellular Hallmark: Chromosomal Instability and DNA Crosslink Sensitivity

At the cellular level, all FA patients share a unique and diagnostically pathognomonic feature: spontaneous chromosomal instability and extreme hypersensitivity to ICL-inducing agents such as diepoxybutane and mitomycin C [44,45].

When FA cells are exposed to bifunctional DNA cross-linking agents such as diepoxybutane or mitomycin C, they display markedly increased chromosomal fragility, including chromatid/chromosome breaks, gaps, radial figures, and other chromosomal rearrangements, compared with control cells [46,47]. This hypersensitivity forms the basis of the standard diagnostic test for FA as it reflects the impaired ability to repair DNA damage, particularly ICLs and DNA–protein crosslinks (DPCs), the characteristic FA cell defect [48,49,50].

The chromosomal fragility observed in FA cells is not limited to exogenous damage [48] as they accumulate spontaneous DNA damage during normal cellular processes, including DNA replication and transcription [50,51]. This accumulation of endogenous damage is particularly relevant during hematopoietic stem cell (HSC) differentiation, where rapid cellular proliferation and extensive transcriptional reprogramming create conditions of heightened genotoxic stress [51].

The inability to adequately respond to endogenous damage likely contributes significantly to the progressive depletion of the HSC pool observed in FA patients, ultimately culminating in bone marrow failure [51].

A significant advancement in understanding FA pathogenesis came from genetic studies in mice, which demonstrated that the hematologic phenotype of FA involves not only FA genes but also aldehyde metabolism genes (ALDH2 or ADH5) [52,53], which contribute to DNA instability. These observations revealed a crucial genetic interaction between the FA pathway and aldehyde detoxification systems, implicating aldehyde-mediated DNA damage as a key driver of disease manifestations [54].

Aldehydes, particularly formaldehyde and acetaldehyde, are highly reactive molecules capable of forming DPCs and DNA ICL [55,56]. While these compounds can arise from exogenous sources, they are also generated endogenously as obligatory byproducts of normal cellular metabolism [57]. Of relevance is the production of formaldehyde during histone and protein demethylation reactions catalysed by enzymes such as LSD1 and Jumonji-domain-containing demethylases [58].

These epigenetic modifications are essential for transcriptional regulation, since formaldehyde production is linked to the dynamics of gene expression [59]. During processes requiring extensive transcriptional reprogramming—such as hematopoietic differentiation—nuclear formaldehyde levels can rise dramatically [60].

**Figure 1:**
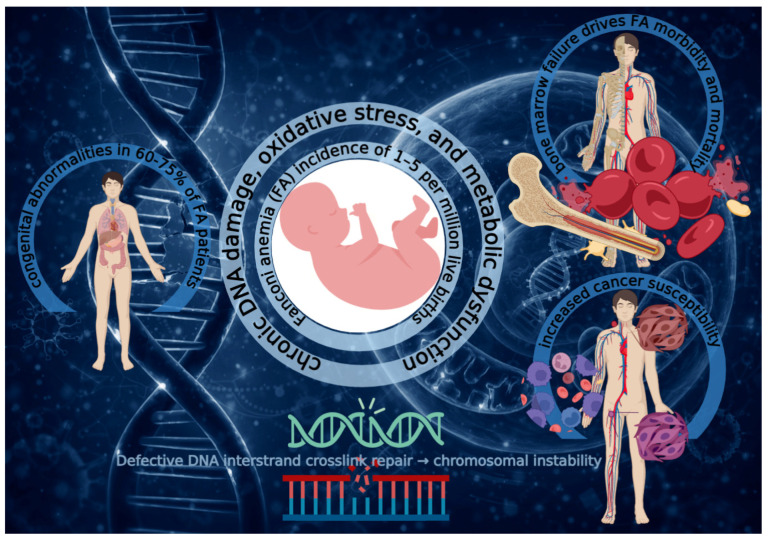
Overview of Fanconi anemia. FA is a rare genetic disorder (1–5 cases per million live births) caused by defects in DNA interstrand crosslink repair, leading to chromosomal instability. FA is associated with chronic DNA damage, oxidative stress, and metabolic dysfunction, and is clinically characterized by congenital abnormalities (60–75% of patients), progressive bone marrow failure, and increased cancer susceptibility.

In cells with intact FA pathway function, this endogenous formaldehyde-induced damage can be efficiently repaired; however, in FA-deficient cells, this damage accumulates, leading to replication fork stalling, DNA breaks, and cell death or senescence [52,61]. This creates a pathogenic feed-forward loop wherein the very processes required for normal cellular differentiation generate DNA damage that cannot be adequately repaired due to the primary FA pathway defect [61,62,63]. The main clinical and molecular features of FA are summarized in Figure 1.

## 2. Beyond DNA Repair: Impact of Altered Mitochondrial Structure and Function in Fanconi Anemia

**Figure 2:**
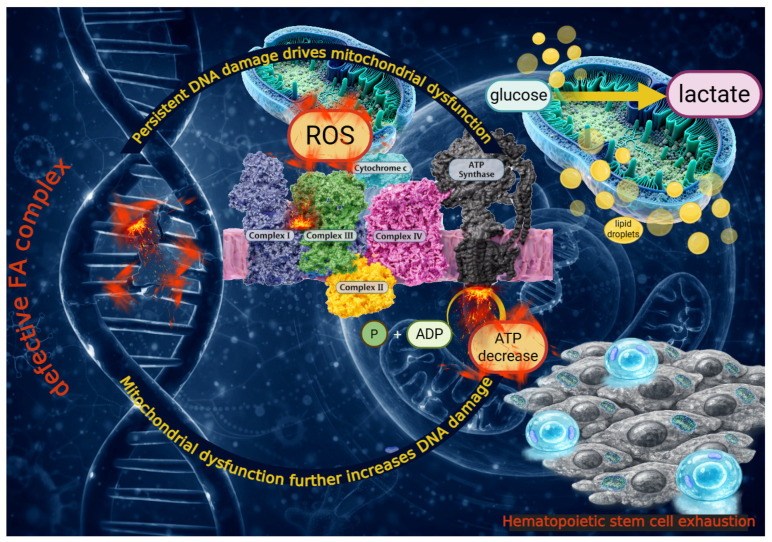
Defective mitochondrial energy function in FA cells. The figure illustrates how a FANC-mutated cell is characterized by mitochondrial impairment and increased ROS production. This dysfunction triggers a metabolic shift from oxidative phosphorylation to glycolysis (glucose to lactate), leading to an ATP decrease, lipid droplet accumulation, and further DNA damage. The cumulative effect results in hematopoietic stem cell exhaustion.

Although FA is classically defined by defective DNA ICL repair and genomic instability, increasing evidence indicates that FA pathophysiology also involves profound mitochondrial functional and structural abnormalities. Literature reports that FA primary cells isolated from patients (i.e., lymphocytes and fibroblasts) and in immortalized cell lines derived from patient cells (i.e., lymphoblasts) exhibit marked metabolic defects, particularly affecting oxidative phosphorylation (OxPhos) and overall cellular bioenergetics [64,65,66,67,68]. This emerging metabolic dimension appears to be functionally connected to the canonical defect in DNA repair, as correction of the mutated FA gene restores not only DNA repair capacity but also metabolic function, suggesting that FA proteins may coordinate genome stability with cellular energy homeostasis. In detail, *FANCA*-mutated cells display inefficient electron transport between complexes I and III associated with the uncoupling between oxygen consumption and ATP synthesis [69], a condition that favors electron leakage and premature oxygen reduction, resulting in increased reactive oxygen species (ROS) production [65] (Figure 2).

Other studies show that FA proteins also appear to regulate cellular energy metabolism more directly. FANCD2 has been shown to modulate mitochondrial ATP production through interaction with ATP5α, linking FA pathway integrity to oxidative phosphorylation efficiency and mitochondrial bioenergetics [70]. Together with evidence for stress-induced mitochondrial localization of FANCD2 and FANCI, these findings support the view that FA proteins contribute not only to genomic stability but also to metabolic homeostasis [70,71,72,73,74,75,76].

Patient-derived data further support the presence of mitochondrial abnormalities, including mtDNA copy number changes, mtDNA sequence variants, and downregulation of mtDNA-encoded respiratory genes [67,77,78]. In healthy cells, *FANCD2* and *FANCI* translocate to mitochondria in response to oxidative stress, where they participate in mitochondrial DNA maintenance and stress-adaptive responses [71,72,73,74]. Conversely, mutations in FA genes impair mitochondrial stress signaling, leading to accumulation of mitochondrial DNA damage, defective organellar quality control, and progressive mitochondrial dysfunction, ultimately contributing to cellular senescence and apoptosis, particularly in HSCs/HPCs [67,75,76].

Consistent with these defects, FA cells exist in a chronically low-energy state, as reflected by a reduced ATP/AMP ratio, and compensate for impaired aerobic metabolism by increasing glycolytic dependence, glucose uptake, and lactate production, representing an adaptive attempt to reprogram metabolism to sustain energy demands [65,69,79] (Figure 2). Other studies suggest that this rewiring is not restricted to glucose metabolism, but extends to glutamine and lipid pathways, supporting a broader state of metabolic inflexibility [80,81]. In this context, mitochondrial dysfunction in FA should not be viewed as an isolated respiratory defect, but rather as a system-level alteration that impairs the cell’s ability to adapt energy production under stress [7,65,80,82,83], thereby potentially exacerbating oxidative damage and cellular vulnerability, as antioxidant responses require high levels of energy [84].

Consistently, ultrastructural analyses show abnormal mitochondrial morphology in FA cells, including swelling, cristae disorganization, reduced mitochondrial volume, and increased fragmentation [85,86,87]. Three-dimensional reconstructions show an increased number of mitochondria with significantly reduced average volume in *FANCA*- and *FANCC*-deficient cells, consistent with excessive mitochondrial fragmentation [85]. These structural abnormalities are not purely morphological but also have direct functional consequences on mitochondrial efficiency since small mitochondria with few cristae are less efficient in ATP production [88].

Interestingly, this fragmented mitochondrial phenotype reflects an imbalance in mitochondrial dynamics. In FA cells, the fission GTPase DRP1 is overexpressed and aberrantly recruited to the mitochondrial surface while the fusion machinery—regulated by mitofusin-2 (MFN2) at the outer mitochondrial membrane and optic atrophy-1 (OPA1) at the inner membrane—fails to counteract excessive fission [85,89]. In addition, DRP1 activity and mitochondrial recruitment are tightly regulated by post-translational modifications, including phosphorylation events controlled by stress-responsive and energy-sensing pathways [90], suggesting that the metabolic imbalance observed in FA cells may directly promote excessive mitochondrial fission. Specifically, a fragmented mitochondrion resulting from the excessive expression of fission-related proteins (e.g., DRP1) relative to fusion-associated proteins not only undergoes structural alterations but also functional ones, leading simultaneously to reduced ATP production and increased ROS generation, incrementing the oxidative damage in FA cells [89].

Simultaneously, reduced expression of mitophagy and autophagy regulators, including Parkin and Beclin-1, impairs the clearance of damaged mitochondria, promoting their accumulation, thereby perpetuating mitochondrial dysfunction and amplifying oxidative stress [85,89,91,92]. In fact, under healthy conditions, Parkin is recruited to damaged or depolarized mitochondria through the PINK1–Parkin pathway. Once activated, Parkin ubiquitinates proteins on the outer mitochondrial membrane, marking dysfunctional mitochondria for selective removal via mitophagy, which is sustained by several proteins, including Beclin-1, a core component of the class III PI3K complex that is essential for autophagosome nucleation, thereby enabling the initiation of autophagy and mitophagy processes [93,94]. Interestingly, in Indian FA cohorts, deregulation of mitophagy-related genes has been observed, which may contribute to bone marrow failure [67]. These alterations are not merely secondary to generalized cellular stress but appear to be directly linked to FA protein function [92,95], supporting a direct link between mitochondrial quality control defects and FA pathophysiology.

In addition, FA cells also exhibit endoplasmic reticulum stress, impaired calcium homeostasis, and cytoskeletal disorganization, which further contribute to mitochondrial dysfunction and cellular fragility [64,96].

However, it is important to note that these metabolic alterations have been confirmed in immortalized cellular models and, in some cases, in primary cells isolated from patients, whereas only limited data are available from animal models. This discrepancy is partly due to the fact that FA animal models do not fully recapitulate the human disease. Indeed, FA animal models are not commonly used to validate in vitro findings, as they fail to reproduce key clinical features such as spontaneous bone marrow failure [54] and therefore cannot reliably support the clinical translation of these findings. 

## 3. Oxidative Stress and Impaired Redox Homeostasis in Fanconi Anemia

### 3.1. Mitochondrial ROS–Driven Macromolecular Damage in Fanconi Anemia

Considering that defective mitochondrial function and quality control represent one of the major sources of ROS [66,97,98,99], FA cells are characterized by an accumulation of oxidative damage [100,101]. Chronic exposure of FA cells to elevated mitochondrial-derived ROS results in progressive oxidation of nucleic acids, lipids, and proteins, ultimately compromising cellular integrity and function [51,82]. Among these targets, DNA is particularly vulnerable, as FA patients and cellular models exhibit significantly increased levels of oxidative DNA lesions, most notably 8-hydroxy-2′-deoxyguanosine (8OHdG) [3,5,102]. In addition, oxidative DNA damage in FA seems not only globally increased but may also affect functionally relevant genomic regions. In particular, FA pathway deficiency has been linked to oxidative damage and transcriptional dysregulation at promoters of antioxidant defense genes, thereby impairing adaptive stress responses [103,104].

In parallel, chronic oxidative stress promotes extensive lipid peroxidation, as indicated by increased levels of malondialdehyde (MDA), 4-hydroxynonenal (4-HNE), and other lipid peroxidation products in FA cells and patient-derived samples [82,83,105]. In detail, oxidative degradation of polyunsaturated fatty acids leads to the formation of highly reactive aldehydes, including MDA, 4-HNE, and acrolein, which can diffuse and form covalent adducts with DNA and proteins [106]. These secondary reactive species exacerbate oxidative damage; for example, MDA is mutagenic in human cells [107], while lipid peroxidation-derived aldehydes such as crotonaldehyde, acrolein, and 4-HNE are relevant sources of DNA-reactive damage, including ICL [108]. The accumulation of lipid peroxidation–derived ICLs is particularly harmful in the context of FA, since they represent lesions that FA cells are intrinsically inefficient at repairing [105]. Thus, lipid peroxidation acts as a critical amplifier of genomic instability, linking oxidative stress to the core DNA repair defect.

Protein oxidation represents an additional layer of oxidative damage driven by persistent ROS exposure. Increased protein carbonylation, a hallmark of oxidative protein modification, has been consistently reported in FA cells [82,83,109] and reflects irreversible oxidative alterations of enzymes, structural proteins, and signaling mediators. Such modifications impair enzymatic activity, disrupt protein–protein interactions, and compromise essential cellular processes, thereby exacerbating metabolic dysfunction and stress sensitivity.

Together, these observations support a model in which mitochondrial dysfunction–driven ROS production in FA initiates and sustains a cascade of oxidative damage affecting all major classes of macromolecules. This cumulative oxidative burden establishes a pathogenic feed-forward loop, wherein mitochondrial impairment fuels oxidative injury, and oxidative damage further destabilizes mitochondrial and cellular homeostasis, ultimately accelerating disease progression.

### 3.2. Causes of Defective Adaptive Antioxidant Responses in Fanconi Anemia

In the context of chronic mitochondrial-derived ROS production and cumulative oxidative damage, an equally critical pathogenic feature of FA is the failure to activate an effective adaptive antioxidant response. Despite sustained exposure to oxidative stress, FA cells do not appropriately activate detoxifying and redox-buffering systems, resulting in a persistent imbalance between ROS generation and cellular defense mechanisms [7,68,82]. This defective adaptation represents a key factor that allows oxidative damage to accumulate and propagate across cellular compartments [5,51,100].

Early studies investigating antioxidant enzyme activity in FA produced seemingly paradoxical results. Literature reported increased activities of manganese superoxide dismutase, catalase, and glutathione peroxidase in FA fibroblasts, proposing that this reflected a compensatory response to chronic oxidative stress [110,111]. However, subsequent work has clarified that this apparent upregulation is highly context-dependent and does not translate into a robust or sustained antioxidant capacity [6,101,111]. More recent and comprehensive analyses have instead revealed a generalized downregulation of antioxidant defenses in multiple FA cell types [82,103,109]. In detail, FA lymphoblasts and fibroblasts exhibit significantly reduced expression of key detoxifying enzymes, including catalase and glutathione reductase, compared with gene-corrected counterparts [82]. Importantly, this downregulation was linked to epigenetic alterations, specifically hypoacetylation of chromatin regions controlling antioxidant gene expression, suggesting that transcriptional repression rather than enzyme instability underlies the impaired response [82]. These findings are in line with transcriptomic and promoter-level analyses showing broad suppression of antioxidant genes and selective accumulation of oxidative DNA lesions at their promoters in FA bone marrow [103,112], and collectively indicate that FA cells fail to transcriptionally reprogram antioxidant pathways in the face of oxidative challenge.

Tissue-specific analyses further support the concept of defective stress adaptation rather than uniform antioxidant insufficiency. Studies in FA red blood cells carrying the common *FANCA* c.295C>T mutation revealed reduced catalase activity at baseline, while glutathione peroxidase and GSTP1 activities were paradoxically elevated, consistent with a preconditioned oxidative stress state [6]. Critically, upon exposure to exogenous stressors such as diepoxybutane or prolonged culture conditions, FA red blood cells exhibited a marked decline in GSTP1 and GPx activities, in contrast to control cells that maintained or increased enzyme activity [6,82]. This inability to sustain or augment antioxidant defenses upon stress exposure highlights a fundamental defect in adaptive capacity and is consistent with broader observations that FA cells fail to upregulate antioxidant enzymes in response to oxidative challenge [82].

Central to this impaired response is dysregulation of the glutathione system, the primary intracellular redox buffer. FA cells display variable total glutathione levels depending on cell type and disease stage; however, a disturbed GSH/GSSG ratio is a consistent feature, reflecting an oxidized intracellular environment [101,104]. In parallel, downregulation or insufficient activity of glutamate–cysteine ligase, the rate-limiting enzyme for de novo glutathione synthesis, likely due to the decreased ATP levels, further limits the capacity to replenish GSH under sustained oxidative stress, in line with the concept that enzymatic control of the glutathione shunt is a key determinant of redox state [101,113]. Together, these alterations constrain the ability of FA cells to restore redox balance once oxidative stress is initiated and help explain the persistent pro-oxidant state documented in blood and bone marrow compartments of FA patients [8,101,104].

A major mechanistic explanation for this failure lies in impaired activation of the NRF2–KEAP1 signaling axis, the master regulator of antioxidant gene expression [114]. Paradoxically, despite elevated ROS levels, FA cells fail to adequately stabilize and activate NRF2, in part because components of the FA pathway themselves modulate KEAP1–NRF2 signaling; for example, PALB2/FANCN competes with NRF2 for KEAP1 binding and promotes NRF2 nuclear accumulation and antioxidant function [115]. Du and colleagues provided a mechanistic insight by demonstrating that FA proteins cooperate with the chromatin remodeler BRG1 to protect antioxidant gene promoters from oxidative DNA damage [103]. Under oxidative stress, FA proteins are recruited to these promoters, facilitating BRG1 binding and preserving transcriptional competence [103,116]. In FA-deficient cells, this protective mechanism collapses, leading to preferential accumulation of oxidative lesions within promoter regions of NRF2 target genes and heightened nuclease hypersensitivity [103]. Consequently, key antioxidant genes—including NQO1, HO-1, GCLC, and multiple glutathione S-transferases—exhibit persistently reduced transcription in FA bone marrow and primary cells [101,103]. Notably, this repression is driven mainly by increased initial oxidative damage at these promoters, rather than grossly impaired repair kinetics, establishing a self-reinforcing loop in which oxidative stress disables the very genes required for its resolution [101,103].

Recent work has further shown that epigenetic dysregulation synergizes with oxidative promoter damage to silence antioxidant programs in FA [82]. Hypoacetylation of detoxifying gene loci contributes to stable transcriptional repression, which can be partially reversed by histone deacetylase inhibition [82,117]. Treatment with valproic acid (VPA) restored catalase and glutathione reductase expression, reduced lipid peroxidation, corrected metabolic defects, and improved mitomycin C resistance, underscoring the functional relevance of epigenetic control in FA redox biology [82]. Consistently, other HDACi such as vorinostat modulate epigenetic regulators and reduce chromosomal breakage in FA cells, supporting the therapeutic potential of targeting chromatin state in this disease [117].

Beyond transcriptional regulation, several FA proteins play direct roles in redox control. PALB2/FANCN directly interacts with the oxidative stress sensor KEAP1 via an ETGE motif, competing with NRF2, promoting NRF2 nuclear accumulation and function, and lowering intracellular ROS levels [115]. FANCG localizes to mitochondria and binds the mitochondrial peroxidase peroxiredoxin 3 (PRDX3); loss of FANCG or related FA proteins leads to PRDX3 mislocalization, reduced thioredoxin-dependent peroxidase activity, distorted mitochondrial morphology, and heightened sensitivity to H_2_O_2_ and mitomycin C, highlighting its role in mitochondrial antioxidant defense [111,118]. FANCC, FANCA, and FANCG participate in detoxification pathways through interactions with NADPH cytochrome P450 reductase, cytochrome P450 2E1, and glutathione S transferase P1 1, linking the FA core complex to xenobiotic metabolism and ROS handling [101,103,110,118]. FANCJ/BACH1 further connects oxidative base damage sensing to repression of heme oxygenase 1 and other antioxidant response element–driven genes, integrating redox signaling with transcriptional control [101].

**Figure 3:**
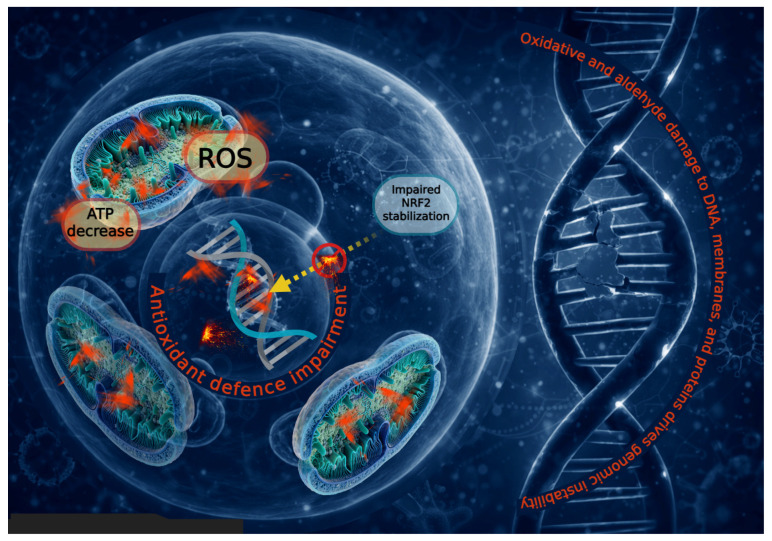
Failed antioxidant response to oxidative stress in Fanconi Anemia cells. The figure shows that the overproduction of ROS and mitochondrial dysfunction are not counteracted by a proper antioxidant response due to impaired NRF2 stabilization. This imbalance results in impaired antioxidant defense, leaving the cell vulnerable to oxidative and aldehyde damage. The resulting accumulation of lesions in DNA, membranes, and proteins significantly increases the risk of genomic instability.

Collectively, these findings establish that FA is characterized not only by excessive oxidative stress but also by a profound inability to respond to it. The convergence of transcriptional, epigenetic, and protein-level defects in antioxidant regulation allows oxidative damage to persist, amplifies mitochondrial and genomic instability, and accelerates disease progression. It is important to note that, as observed for metabolic alterations, the most prominent findings have also been obtained in immortalized cellular models. A schematic overview of the impaired antioxidant response in FA cells is provided in Figure 3, and Table 1 reports the principal alterations involved in oxidative stress production and the accumulation of oxidative damage in FA cells, indicating increases and decreases compared with control cells.

### 3.3. Potential Clinical Relevance of Redox Biomarkers in Fanconi Anemia

Emerging evidence suggests that oxidative stress–related biomarkers may provide clinically relevant adjunctive information in FA, although they are not yet validated for routine clinical use. For example, as a consequence of [6] oxidative environment, approximately 68% of FA patients display elevated red cell distribution width, which correlates with the severity of hematological impairment, including anemia, thrombocytopenia, and neutropenia, and may serve as a simple, accessible indicator of bone marrow failure progression [120]. In addition, alterations in erythrocyte antioxidant systems have been reported in FA, including a compensatory increase in glutathione peroxidase and glutathione S-transferase activity alongside reduced catalase activity in patients carrying the homozygous FANCA c.295C>T variant, reflecting a dynamic but fragile adaptive response that appears to collapse under increased oxidative stress and correlates with disease progression [6].

At the molecular level, increased levels of oxidative DNA damage markers such as 8OHdG, elevated pro-oxidant signatures in circulating and cellular compartments, and altered antioxidant enzyme activities in erythrocytes and other blood-derived cells have been consistently reported in FA patients [3,4,5,6,104,121].

Collectively, these findings indicate that redox-related biomarkers are measurable in patient-derived samples and may reflect underlying cellular stress and metabolic dysregulation. However, their clinical utility is currently constrained by substantial inter-individual variability, cell-type and oxygen-dependent metabolic effects, and the absence of standardized longitudinal studies linking biomarker dynamics to clinically meaningful outcomes such as bone marrow failure progression, clonal evolution, or therapeutic response [6,7,104].

Therefore, while oxidative stress markers may hold promise for future patient stratification and biological monitoring in FA, they should presently be considered exploratory translational tools rather than established clinical surrogates for disease monitoring or treatment decision-making.

### 3.4. Oxygen-Dependent Metabolic Vulnerability in Fanconi Anemia

A distinctive feature of FA is the strong context dependency of its metabolic phenotype, particularly in relation to oxygen availability. Several studies have demonstrated that metabolic abnormalities and oxidative stress in FA cells are largely unmasked when hematopoietic cells transition from the hypoxic bone marrow niche to the normoxic peripheral circulation [7,51,122]. The bone marrow microenvironment is characterized by low oxygen tension, with direct intravital measurements in mice showing local pO_2_ values commonly around 1–5% O_2_ (≈9–32 mmHg), with the lowest levels (~1.3% O_2_) in deeper perisinusoidal regions [123,124,125,126,127]. By contrast, peripheral blood and most tissues are exposed to near-atmospheric oxygen levels of approximately 20% O_2_, which are routinely used in standard cell culture conditions [7,128,129]. Within this hypoxic niche, FA HSCs/HPCs exhibit metabolic profiles broadly comparable to those of healthy controls: both FA and control bone marrow mononuclear cells maintained under 3–5% O_2_ rely predominantly on anaerobic glycolysis, show low mitochondrial oxidative phosphorylation, and generate only modest levels of ROS [7]. Under these conditions, FA bone marrow cells do not display the marked defects in oxidative phosphorylation, redox imbalance, or G2/M arrest that become evident after exposure to normoxia, supporting the view that the hypoxic bone marrow niche provides a relatively “safe” metabolic environment for FA HSCs/HPCs [7,125,126,127].

Upon exposure to normoxic conditions, however, FA cells fail to appropriately activate OxPhos and instead develop pronounced mitochondrial dysfunction, progressive energy depletion, and excessive accumulation of oxidative damage [65,82,83]. This transition is accompanied by a sharp increase in ROS production, lipid peroxidation, and oxidative DNA lesions, including 8OHdG, documented in FA patient leukocytes and cellular models [5,82,100,101]. Crucially, FA cells display an intrinsic inability to counterbalance this oxidative burden due to defective induction and activity of key antioxidant defense systems. Reduced expression and activity of enzymes such as catalase, superoxide dismutase, glutathione peroxidase, glutathione reductase, and aldehyde dehydrogenase in FA lymphoblasts, fibroblasts, bone marrow mononuclear cells, and red blood cells, together with a failure to upregulate these pathways in response to oxidative insults such as hydrogen peroxide or normoxic shift, likely underlie this vulnerability [7,68,82,83,101,121].

As a consequence, the metabolic stress associated with differentiation, mobilization, and exposure to higher oxygen tension generates a pathological feed-forward loop in FA cells, in which mitochondrial dysfunction amplifies oxidative stress, and oxidative stress further impairs mitochondrial and genomic integrity [7]. ROS directly induce DNA base modifications and strand breaks, while lipid peroxidation-derived aldehydes generate DNA ICLs, the hallmark lesions that FA cells are inherently unable to repair efficiently [130,131].

Thus, the protective hypoxic bone marrow niche transiently buffers FA HSC from oxidative and metabolic stress, whereas exit from this environment unmasks a latent metabolic vulnerability [7]. Over time, repeated exposure to normoxic conditions contributes to cumulative cellular damage, HSC attrition, and progressive bone marrow failure in FA [7,132].

## 4. Revisiting the Role of Oxidative Stress in Fanconi Anemia: Secondary Driver and Bidirectional Amplifier of Disease Pathogenesis

Although impaired mitochondrial function and oxidative stress are consistently observed across multiple FA models and patient-derived systems [65,66,68], their precise role in disease pathogenesis requires careful contextualization.

Fanconi anemia is fundamentally defined by a primary defect in DNA interstrand crosslink repair, which represents the initiating molecular lesion [31,133,134]. Within this framework, redox and metabolic alterations should not be interpreted as a uniform upstream cause of disease, but rather as context-dependent processes that may arise downstream of DNA repair deficiency and, in turn, contribute to disease amplification.

From a mechanistic standpoint, defective FA/BRCA pathway activity leads to the accumulation of endogenous DNA lesions and persistent cellular stress responses [32]. These alterations can indirectly impair mitochondrial function through multiple routes, including replication stress signaling, altered metabolic regulation, and activation of stress-responsive pathways. Mitochondrial dysfunction is therefore commonly observed in FA systems [65,66,68]; however, whether it represents a direct consequence of FA protein loss or a secondary adaptive response to chronic genotoxic stress remains incompletely resolved.

In turn, mitochondrial impairment is a well-established source of increased ROS production [97,98,135,136], providing a plausible downstream mechanism for the oxidative phenotype observed in FA. This positions oxidative stress, at least in part, as a secondary consequence of the primary DNA repair defect. Importantly, however, this interpretation is supported mainly by correlative and model-dependent evidence, and a fully defined causal chain linking FA gene loss to mitochondrial dysfunction has not yet been established across all cellular contexts.

Conversely, accumulating evidence supports a reverse contribution, whereby ROS and lipid peroxidation–derived aldehydes can induce additional DNA lesions, including ICL and oxidative base damage. These lesions are particularly detrimental in FA cells, which are intrinsically deficient in repairing DNA interstrand crosslinks. This establishes a mechanistic scenario in which oxidative stress can actively reinforce the primary genomic instability phenotype [82,100,137].

Taken together, current evidence supports multiple, partially overlapping mechanistic frameworks rather than a single linear pathway. One model places oxidative stress predominantly downstream of FA pathway dysfunction, as a consequence of impaired mitochondrial homeostasis. A second model suggests that oxidative stress may independently contribute to DNA damage accumulation and, thus, act as a co-driver of disease progression. A third, non-mutually exclusive framework proposes that mitochondrial dysfunction and oxidative stress emerge from shared stress-response networks activated by persistent DNA damage, rather than from a strictly hierarchical cause–and–effect relationship.

**Figure 4:**
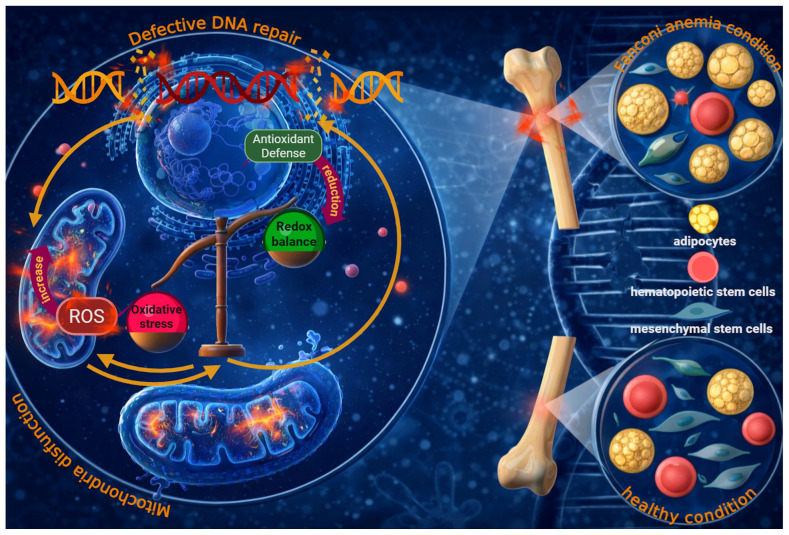
Integrated conceptual model linking defective DNA repair, mitochondrial dysfunction, oxidative stress, and hematopoietic stem cell impairment in FA. DNA repair defects in FA lead to mitochondrial alterations, resulting in increased ROS production and impaired oxidative phosphorylation. The consequent redox imbalance and insufficient antioxidant response—an energy-demanding process—further amplify oxidative stress, creating a self-reinforcing cycle of cellular damage. This systemic metabolic and redox dysregulation ultimately contributes to the dysfunction of bone marrow niches, promoting adipocyte accumulation and impairing hematopoietic stem and progenitor cell maintenance, as illustrated by the comparison between FA and healthy conditions.

Rather than representing a linear cascade, these processes are better conceptualized as a systems-level network in which genomic instability, metabolic dysfunction, and redox imbalance are dynamically interconnected through multiple feedback loops (Figure 4).

Within this network, different nodes may dominate depending on the cellular context. For example, in highly proliferative compartments such as hematopoietic progenitors, replication stress and DNA damage signaling may act as primary drivers, whereas in metabolically active or oxygen-exposed environments, mitochondrial dysfunction and ROS production may play a more prominent role [7].

Despite these distinctions, the most consistent integrative interpretation is that oxidative stress functions predominantly as an amplifying and propagating factor rather than a primary initiating event. In this context, it establishes a feed-forward loop that reinforces genomic instability, metabolic dysfunction, and stem cell attrition. Importantly, the relative contribution of each mechanism likely varies across FA complementation groups, tissue types, developmental stages, and environmental conditions such as oxygen availability.

A key unresolved question remains whether oxidative stress represents exclusively a downstream consequence of FA pathway deficiency or whether it can, under specific conditions, contribute to disease initiation by promoting early DNA damage before overt genomic instability is established. Addressing this issue will require longitudinal and causality-oriented experimental approaches, as current evidence is largely derived from cross-sectional or model-dependent observations.

## 5. Molecular and Clinical Consequences of Metabolic and Redox Dysfunction in Fanconi Anemia

The convergence of mitochondrial metabolic defects and failure of adaptive antioxidant responses has profound molecular and clinical consequences in FA, particularly affecting tissues and cell populations with high metabolic demands or intrinsic sensitivity to oxidative stress. Among these, HSCs represent the most vulnerable compartment and account for the hallmark manifestation of bone marrow failure [16,51,138,139,140]. Under physiological conditions, HSCs reside in hypoxic bone marrow niches and rely primarily on glycolytic metabolism to preserve quiescence and long-term self-renewal [125,126,141]. FA-associated metabolic and redox abnormalities critically disrupt this finely tuned balance, as FA HSCs and HPCs exhibit heightened oxidative stress, replication and DNA damage stress responses, altered mitochondrial metabolism and mitophagy, and progressive attrition leading to bone marrow failure [7,8,16,142]. While the FA/BRCA pathway defect remains the initiating lesion, the resulting mitochondrial and redox abnormalities are better viewed not as passive by-products, but as reinforcing mechanisms that can amplify genomic instability and tissue dysfunction through positive feedback loops [122,143,144].

### 5.1. HSC Dysfunction and Bone Marrow Failure

The physiological consequences of this defective adaptive response are particularly evident in HSCs/HPCs. In vivo stress reporter studies using Gadd45β luciferase mice demonstrated that FA-deficient HSPCs (*FANCA*^−^/^−^, *FANCC*^−^/^−^) exhibit a persistent response to oxidative stress, with prolonged activation of oxidative stress–responsive transcription and DNA damage signaling after challenge, in contrast to transient responses in wild-type cells [100,145,146]. These prolonged checkpoints correlate with the accumulation of unrepaired oxidative DNA lesions in damage-sensitive genes within HSCs/HPCs [100,145]. Importantly, genetic correction of FANCA or FANCC deficiency nearly abolished the long-lasting oxidative stress–induced G2/M arrest and DNA damage response in vivo, restoring normal recovery dynamics and underscoring the essential role of the FA pathway in terminating oxidative stress responses in HSCs/HPCs [100,145,147,148].

Persistent activation of stress signaling pathways, due to chronically elevated ROS, activates DNA damage-response pathways, particularly the p53–p21 axis and TGF β signaling in FA HSPCs, leading to cell cycle arrest, senescence, and increased apoptosis susceptibility [16,149,150]. This, in turn, causes HSC exhaustion, impaired self-renewal, and progressive bone marrow failure [2,16,48,51,139]. FA patients and mouse models show a reduction in HSCs/HPCs numbers, loss of quiescence, excessive apoptosis or senescence under oxidative and inflammatory stress, and eventual collapse of hematopoiesis that can be mitigated by genetic or pharmacologic interventions targeting these stress pathways [16,48,100,139,146,151].

Mitochondrial dysfunction further compounds these defects; normal HSC differentiation requires a metabolic shift from glycolysis to oxidative phosphorylation, but FA HSCs remain locked in a glycolytic state, impairing differentiation and hematopoiesis [7,102]. Chronic oxidative stress also activates inflammatory pathways such as NF-κB and inflammasomes, elevating pro-inflammatory cytokines like TNF-α and IL-6, which damage the bone marrow niche and exacerbate HSC attrition [150,152]. These molecular and cellular dysfunctions collectively contribute to the premature aging phenotype observed in FA patients’ bone marrow [137].

Overall, the interplay of DNA damage responses, metabolic dysregulation, and inflammation underlies the progressive hematopoietic failure characteristic of FA [16,152,153].

### 5.2. Developmental Abnormalities

Metabolic and redox dysregulation may also contribute to the congenital malformations associated with FA [80]. Although direct mechanistic evidence linking these pathways to FA-specific developmental phenotypes remains limited, the available literature supports a biologically plausible contributory role for oxidative stress and mitochondrial dysfunction during embryogenesis [154,155,156,157,158,159]. Developing tissues are particularly sensitive to redox imbalance because morphogenesis depends on tightly regulated developmental pathways, including Sonic hedgehog, BMP, and Wnt signaling, as well as on adequate mitochondrial energy supply to sustain rapidly proliferating progenitor populations [154,155,156,157]. In this context, excess ROS, impaired mitochondrial metabolism, and defective stress adaptation could exacerbate the consequences of the primary FA defect by interfering with developmental signaling, cell survival, and tissue remodeling during critical windows of organogenesis [154,155,158]. Experimental evidence from animal models shows that oxidative stress damages cellular components like DNA, proteins, and lipids, impairing normal morphogenesis and tissue remodeling, which are crucial for proper development [155,158]. Antioxidant treatments in these models reduce the severity of birth defects by restoring redox homeostasis, suggesting that targeting oxidative stress deserves further investigation in developmental contexts [154,159]. Moreover, epidemiological data link higher oxidative balance scores with reduced risk of limb deficiencies and other congenital anomalies, supporting the role of oxidative stress in human developmental defects [159]. Overall, these findings underscore the importance of tightly regulated redox balance during embryonic development and suggest that antioxidant strategies deserve further investigation in relation to FA-related developmental abnormalities [154,155,159].

### 5.3. Cancer Predisposition

While defective ICL repair is a primary determinant of cancer susceptibility in FA, metabolic and redox abnormalities introduce additional oncogenic pressures. Chronic oxidative stress in FA cells leads to the accumulation of mutagenic DNA lesions such as 8OHdG and lipid peroxidation–derived adducts [7], which increase mutational burden due to defective repair capacity [118,134,160]. FA cells exhibit a constitutive reliance on glycolytic metabolism, resembling the metabolic phenotype of many cancers, with altered OxPhos [7] and increased purine biosynthesis that may favor a cellular state permissive for oncogenic transformation [161]. Persistent oxidative stress–induced inflammation promotes a pro-tumorigenic microenvironment, while oxidative stress–driven epigenetic alterations stabilize transcriptional programs favoring proliferation and survival [118,137]. The tissue specificity of FA-associated malignancies—including acute myeloid leukemia, HNSCC, and gynecological cancers—likely reflects differential susceptibility to oxidative and metabolic stress across tissues, with FA HNSCCs showing characteristic genomic instability driven by structural variants and inflammatory signaling [20,79,162]. Overall, the interplay between defective ICL repair, metabolic dysregulation, chronic oxidative stress, and inflammation underlies the heightened cancer risk in FA patients [26,163].

### 5.4. Premature Aging

Mitochondrial dysfunction, oxidative stress, and defective stress adaptation are widely implicated in biological aging and are also recurrent features of FA [164,165,166,167,168]. These shared processes provide a plausible framework for interpreting FA as a syndrome with features of premature aging, although the relative contribution of redox and metabolic mechanisms to this phenotype remains to be defined. Aging is characterized by a decline in ATP production alongside elevated ROS levels and diminished antioxidant capacity, resulting in oxidative damage to DNA, lipids, and proteins [167,169]. This imbalance triggers chronic inflammation and stem cell exhaustion, further accelerating tissue degeneration and age-related diseases [170,171]. Mitochondria thus serve as central hubs regulating oxidative stress and inflammation, linking molecular dysfunctions to systemic aging phenotypes [166,172]. Since the metabolic and redox abnormalities observed in FA are not restricted to hematopoietic tissues [168] and FA patients’ blood is characterized by circulating biomarkers of oxidative stress [6], it is possible to hypothesize that the premature aging phenotype observed in FA patients [173,174] may represent the cumulative outcome of chronic oxidative stress, mitochondrial dysfunction, and stem cell exhaustion.

## 6. Therapeutic Implications

These pathophysiological links prompt the question of whether mitochondrial and redox abnormalities can be therapeutically modulated in FA. In addition to current standard approaches such as hematopoietic cell transplantation for hematologic disease and emerging gene-based strategies in selected settings, several complementary interventions targeting oxidative stress, mitochondrial metabolism, chromatin regulation, or oxygen exposure have shown promising effects in preclinical settings, including cellular, ex vivo, and animal models [7,82,175,176,177,178]. However, early clinical experience with redox- or mitochondria-directed interventions remains limited and currently supports tolerability and biomarker modulation more strongly than durable disease-modifying benefit [119,179]. These approaches should therefore be regarded primarily as investigational adjuncts aimed at mitigating cellular stress or improving metabolic resilience, rather than as replacements for the current therapeutic backbone of FA management.

### 6.1. Antioxidant Treatment

Antioxidant molecules have been extensively investigated as potential treatments for FA due to the central role of oxidative stress in its pathophysiology. In vitro and in vivo studies have shown that antioxidants can reduce ROS levels, improve mitochondrial function, and decrease DNA damage in FA models. For example, in lymphocytes and lymphoblasts mutated for the *FANCA* gene, quercetin, a natural flavonoid antioxidant, has demonstrated efficacy in improving energy metabolism and reducing oxidative stress in FA lymphoblast cell lines; combined with other agents like C75 and rapamycin, it showed additive benefits on mitochondrial function and DNA damage reduction [83]. In addition, quercetin has been proposed in a phase 1 clinical trial in children and young adults with FA, demonstrating good tolerability [179]. In detail, 12 patients were enrolled in a dose-finding phase and 18 in an expansion cohort; quercetin was administered orally twice daily, and a bodyweight-adjusted regimen with a maximum adult daily dose of 5000 mg/day was established as the recommended dose [179]. A subset of patients showed reduced ROS levels in peripheral blood and bone marrow stem cells, whereas hematologic benefit remained exploratory and difficult to interpret because blood counts fluctuate intrinsically in FA [179]. Thus, the main clinical signal of quercetin currently lies in tolerability and biomarker modulation rather than in established long-term disease modification, and quercetin should at present be regarded as an investigational adjunct rather than a component of standard FA management [179]. Histone deacetylase inhibitors like VPA have also been shown to enhance antioxidant enzyme expression (e.g., catalase, glutathione reductase), correct metabolic defects, lower lipid peroxidation, and improve cellular survival under oxidative stress in FA cellular models [82]. N-acetylcysteine (NAC), a glutathione precursor, has been investigated mainly in FA cellular models, where it reduces oxidative stress and can improve selected metabolic or structural abnormalities, although these effects are partial and context-dependent [87,180]. In addition, combined antioxidant treatment including NAC in association with α-Lipoic Acid has been reported to improve genetic stability in FA lymphocytes in vitro [177]. In a small study including children with FA, supplementation with vitamins A, C, and E was associated with reductions in lipid peroxidation markers and improvement in selected antioxidant readouts; however, these results were not FA-specific and did not demonstrate sustained correction of bone marrow failure [119]. Overall, current data suggest that antioxidant approaches can modulate biochemical markers of oxidative damage and, in some cases, yield modest hematologic signals, but they have not yet demonstrated durable disease-modifying efficacy in FA patients [119,179]. Moreover, these strategies appear to exert predominately modulatory rather than clearly disease-modifying effects. While preclinical models consistently support a role for redox modulation, translation to clinical benefit remains limited by small cohorts, early-phase designs, heterogeneous patient backgrounds, and uncertainty regarding optimal treatment duration, bioavailability, and target engagement in vivo [119,179]. Furthermore, targeting redox imbalance in vivo remains intrinsically challenging due to the complexity and compartmentalization of cellular redox networks.

### 6.2. Modulator of Energy Metabolism, Mitochondrial Dynamics and Turnover

Because mTOR signaling regulates mitochondrial function [181,182], mitochondrial dynamics [90], and is aberrantly activated in several FA contexts [183,184], the effect of rapamycin, a specific mTOR inhibitor, has been explored as a metabolic modulator in FA models. In FA lymphoblasts, rapamycin lowers aerobic metabolism and oxidative stress but does not improve overall energy status; it also contributes to reducing DNA double-strand breaks, especially when combined with other metabolic modulators like quercetin [83]. Additionally, rapamycin induces autophagy in *FANCA* mutant cells, which helps reverse overactive Notch1 signaling and stabilizes cell viability after DNA damage, promoting cell proliferation and gene expression [176]. Mechanistically, mTOR inhibition by rapamycin may intersect with the FA pathway by modulating FANCD2 levels and functions related to DNA repair and replication fork recovery, as mTOR and FANCD2 cooperate to maintain genomic stability during replication stress [183,185]. In addition, in FA-deficient head and neck cancer cells, rapamycin reduces hyperactivated mTOR signaling, specifically decreasing phosphorylation of translation regulators 4E-BP1 and ribosomal protein S6, which leads to blocked protein synthesis and sensitizes these cells to nutrient stress [184]. Notably, the current evidence remains preclinical and derives from heterogeneous model systems, including FA lymphoblasts and FA-deficient head and neck cancer cells [83,176,184].

Since mitochondrial dynamics and quality control are disrupted in FA cells, through DRP1 overexpression, excessive fission, and defective mitophagy, targeting this axis has emerged as a mechanistically attractive strategy [85,89,91,92]. In this context, P110, a specific DRP1 inhibitor, has been tested in an FA cellular model and showed partial restoration of mitochondrial function [89]. However, FA cells also display a marked dependence on glycolytic adaptation and poor tolerance of normoxia-associated oxidative phosphorylation stress [7,65]. This suggests that mitochondrial rescue strategies will need to balance improved organellar function against the risk of inadvertently increasing ROS generation in vivo.

Based on review-based mitoprotective proposals rather than direct clinical trial evidence in FA, mitochondrial nutrients such as coenzyme Q10, α-lipoic acid, and carnitine have been suggested as potential adjunctive strategies to mitigate mitochondrial dysfunction and oxidative stress [177,186]. Additionally, thrombopoietin (TPO) agonists have shown potential in rescuing HSC deficits in FA mouse models by modulating mitochondrial metabolism, suggesting another therapeutic strategy targeting mitochondrial function [187].

Recently, Balbi et al. reported that ataluren exerts metabolic effects that extend beyond its canonical role in promoting translational readthrough. In *FANCA*-mutated cells, it modulates mitochondrial bioenergetics by improving the OxPhos efficiency and restoring the ATP/AMP balance without stimulating compensatory glycolysis. The drug also reduces oxidative stress, lowering lipid peroxidation and oxidative DNA damage. Mechanistically, these effects are associated with modulation of the mTOR–DRP1 signaling axis, which influences mitochondrial dynamics and energy expenditure [188].

Overall, evidence supports a multidrug approach combining metabolic modulators and agents targeting mitochondrial biogenesis and dynamics to address the complex pathophysiology of FA. However, most data come from cellular or animal models with limited clinical trial validation so far. Therefore, despite their mechanistic appeal, mitochondria-directed interventions cannot yet be considered clinically actionable components of routine FA management and remain primarily hypothesis-driven translational strategies.

### 6.3. Epigenetic Modulation

The discovery that defective antioxidant responses in FA are partially driven by epigenetic repression has opened new therapeutic avenues. Bertola et al. demonstrated that FA cells exhibit hypoacetylation of genes encoding key antioxidant and detoxifying enzymes, including catalase and glutathione reductase [82]. Treatment with histone deacetylase inhibitors (HDACi), particularly VPA, restored histone acetylation, increased antioxidant enzyme expression and activity, reduced lipid peroxidation, and corrected metabolic defects [82]. Importantly, VPA treatment also improved cell survival following exposure to mitomycin C, suggesting that epigenetic reactivation of endogenous defense mechanisms can partially compensate for FA-associated vulnerabilities [82].

Literature also reports that FA cells exhibit decreased expression of genes involved in epigenetic regulation and hypomethylation of tumor suppressor gene promoters, which may promote oncogenesis [117]. Treatment with histone deacetylase inhibitors like Vorinostat has demonstrated promising results in vitro by modulating epigenetic gene expression, reducing chromosomal breaks induced by DNA cross-linking agents, and inducing apoptosis in FA cells, suggesting potential therapeutic benefits [117]. Additionally, vitamin D, through its epigenetic modulation of immune responses, may influence disease progression, highlighting the importance of individual vitamin D status in FA patients [189].

Another epigenetic target is PRMT5; its inhibition reduces the expression of FA genes involved in DNA repair and sensitizes tumor cells to DNA-damaging agents, indicating a possible strategy for chemosensitization in cancers related to FA pathways [190].

Overall, epigenetic therapies may complement antioxidant and metabolic interventions by reactivating endogenous defense programs and modulating FA-related stress responses [82,117,191].

### 6.4. Hypoxic Culture and Oxygen Management

The sensitivity of FA cells to oxygen tension has important therapeutic implications. Early work demonstrated that culturing FA fibroblasts under hypoxic conditions (around 5% O_2_) restores their growth and reduces cell cycle abnormalities caused by elevated oxygen levels, indicating increased nuclear susceptibility to ambient oxygen in FA cells [192]. More recent studies in mouse models of FA (*FANCA* and *FANCC* knockouts) showed that collecting and processing HSCs/HPCs under hypoxia (3% O_2_) significantly increases the number of long-term HSCs compared to normoxic conditions, improving their clonogenic capacity and functional potential [178]. This approach reduces oxidative stress and genomic instability, which are key contributors to BMF in FA [100,193]. The strategy has been successfully applied to enhance ex vivo expansion and preservation of FA HSCs prior to gene therapy or transplantation, supporting its translational relevance [147,178]. While systemic modulation of tissue oxygenation as a clinical intervention remains speculative, these findings highlight how environmental factors like oxygen exposure interact with intrinsic metabolic and redox defects in FA, offering potential avenues for therapeutic exploitation [139,140].

### 6.5. Clinical Feasibility and Translational Limitations of Redox and Mitochondria-Targeted Therapies in FA

Despite the strong mechanistic rationale supporting antioxidant, mitochondrial, and metabolic interventions in FA, their clinical feasibility remains limited and must be interpreted in the context of current standard-of-care management. At present, FA treatment is primarily based on hematopoietic cell transplantation for hematologic manifestations and on supportive care strategies aimed at managing bone marrow failure, endocrine complications, and solid tumor risk, while gene therapy approaches are still emerging in selected investigational settings [119,177,179]. In contrast, most redox- and mitochondria-targeted interventions—including antioxidant supplementation, mTOR modulation, and mitochondrial dynamics regulators—remain at a pre-clinical level [82,83,89]. In these settings, the main reported outcomes are safety, tolerability, and partial modulation of oxidative or metabolic biomarkers, whereas robust and sustained disease-modifying effects have not yet been demonstrated.

From a clinical perspective, several limitations further constrain their applicability. First, antioxidant therapies may present variable bioavailability, dose-dependent effects, and potential interference with physiological redox signaling, raising concerns about long-term systemic use. Second, excessive suppression of ROS may be insufficient to correct the primary DNA repair defect in FA and could theoretically disrupt essential redox-dependent signaling pathways. Third, mitochondrial-targeted strategies face additional challenges, including tissue-specific drug delivery, metabolic compensation (e.g., glycolytic reprogramming), and the risk of exacerbating oxidative stress under certain oxygen conditions.

Similarly, agents acting on mitochondrial dynamics or autophagy pathways may have pleiotropic effects that are difficult to predict in vivo, particularly in a disease characterized by profound genomic instability and bone marrow vulnerability.

Importantly, no mitochondria- or antioxidant-directed intervention has yet been incorporated into standard FA clinical practice as a disease-modifying therapy. Their current role should, therefore, be considered strictly adjunctive and investigational, with potential utility primarily as supportive strategies aimed at reducing cellular stress or improving metabolic resilience.

Ongoing and future clinical studies will be essential to define optimal dosing, treatment duration, long-term safety, and patient subgroups most likely to benefit, as well as to determine whether early biomarker improvements can translate into clinically meaningful outcomes such as sustained hematologic stabilization or reduced clonal evolution risk.

## 7. Conclusions and Future Directions

Fanconi anemia should still be defined primarily as a disorder of defective DNA interstrand crosslink repair. However, the literature reviewed here indicates that mitochondrial dysfunction, oxidative stress, and defective antioxidant responses are recurrent features of FA across several experimental systems and in selected patient-derived studies [3,4,5,6,7,64,65,66,67,68,82,83,100,101,102,103,104,105,106,107,108,109,110,112,144,145]. Rather than replacing the canonical DNA repair framework, these alterations are better interpreted as interconnected modifiers and amplifiers of disease biology, capable of reinforcing genomic instability, stress signaling, and stem cell attrition through bidirectional feed-forward loops [7,51,70,72,76,92,144]. This integrative model may help explain why tissues with high metabolic demands, changing oxygen exposure, or limited stress tolerance, particularly HSCs/HPCs, are especially vulnerable in FA [7,100,138,139,140,145]. From a translational perspective, antioxidant and metabolic interventions have shown encouraging preclinical results and limited early clinical signals, although the current evidence remains insufficient to support disease-modifying claims. Importantly, this framework should be considered an evidence-based integrative model rather than a fully resolved causal sequence, as the relative contribution of mitochondrial and redox alterations likely varies across FA complementation groups, tissues, and experimental systems. Future work should prioritize model-to-patient integration, longitudinal validation of redox biomarkers, clarification of heterogeneity across FA complementation groups, and rational combination strategies that complement, rather than replace, gene-based and hematologic therapies [6,83,101,175,179].

## Figures and Tables

**Table 1:** Overview of oxidative stress and mitochondrial dysfunction markers in Fanconi anemia (FA) cellular and biological models compared with controls.

Marker	Experimental Model	Result in FA vs. Controls	Biological Relevance	References
**Intracellular ROS**	FA lymphoblasts, lymphocytes, and fibroblasts	↑ 1.5–3-fold	Global oxidative stress	[7,68]
**Malondialdehyde (MDA)**	Plasma/FA lymphoblasts and fibroblasts	↑ 1.5–2.5-fold	Lipid damage	[7,82,119]
**8-hydroxy-2′-deoxyguanosine (8OHdG)**	Plasma/FA lymphoblasts, lymphocytes,	↑ 2–4-fold	Oxidative DNA damage	[4,102,104]
**Mitochondrial membrane** **potential (ΔΨm)**	FA lymphoblasts and fibroblasts	↓ 1.5–2 folds	OxPhos activity	[68]
**ATP synthesis**	FA lymphoblasts, lymphocytes, and fibroblasts	↓ 5–10 folds when activated with pyruvate plus malate	OxPhos activity	[7,65,102]
**Oxygen consumption rate (OCR)**	FA lymphoblasts, lymphocytes, and fibroblasts	↓ 3–8 folds when activated with pyruvate plus malate	OxPhos activity	[7,65,102]
**OxPhos efficiency (P/O ratio)**	FA lymphoblasts, lymphocytes, and fibroblasts	↓ 1.5–2 folds when activated with pyruvate plus malate	OxPhos activity	[7,65,102]
**ATP/AMP**	FA lymphoblasts, lymphocytes, and fibroblasts	↓ 2–3.5 folds	Cellular energy status	[7,69]
**Catalase activity**	FA lymphoblasts, lymphocytes	↓ 2.5–3.5 folds	Antioxidant defense	[7,82]
**Glutathione reductase activity**	FA lymphoblasts, lymphocytes	↓ 2.5–3.5 folds	Antioxidant defense	[7,82]
**GSH/GSSG ratio**	FA lymphocytes	↓ 1.5–2.5 folds	Antioxidant capacity	[104]

The table summarizes evidence from multiple experimental systems, including lymphoblasts, lymphocytes, fibroblasts, and plasma, highlighting consistent increases in oxidative stress markers (e.g., ROS, MDA, 8-OHdG) and decreases in mitochondrial function, oxidative phosphorylation efficiency, energy status, and antioxidant defenses in FA cells. Arrows indicate direction of change relative to controls (↑ increase; ↓ decrease), and fold changes are reported where available.

## Data Availability

No new data were created or analyzed in this study.
